# Optimizing the geodetic networks based on the distances between the net points and the project border

**DOI:** 10.1038/s41598-021-04566-0

**Published:** 2022-01-13

**Authors:** Ahmed A. G. AbdAllah, Zhengtao Wang

**Affiliations:** 1 School of Geodesy and Geomatics, Wuhan University, Wuhan, 430079 Hubei China; 2 Faculty of Engineering, Al-Azhar University, Qena, Egypt

**Keywords:** Solid Earth sciences, Engineering

## Abstract

Geodetic networks are important for most engineering projects. Generally, a geodetic network is designed according to precision, reliability, and cost criteria. This paper provides a new criterion considering the distances between the Net Points (NPs) and the Project Border (PB) in terms of Neighboring (N). Optimization based on the N criterion seeks to relocate the NPs as close as possible to PB, which leads to creating shorter distances between NPs or those distances linking NPs with Target Points (TPs) to be measured inside PB. These short distances can improve the precision of NPs and increase the accuracy of observations and transportation costs between NPs themselves or between NPs and TPs (in real applications). Three normalized N objective functions based on L1, L2, and L∞‒norms were formulated to build the corresponding N optimization models, NL1; NL2; and NL∞ and to determine the best solution. Each model is subjected to safety, precision, reliability, and cost constraints. The feasibility of the N criterion is demonstrated by a simulated example. The results showed the ability of NL∞ to determine the safest positions for the NPs near PB. These new positions led to improving the precision of the network and preserving the initial reliability and observations cost, due to contradiction problems. Also, N results created by all N models demonstrate their theoretical feasibility in improving the accuracy of the observations and transportation cost between points. It is recommended to use multi-objective optimization models to overcome the contradiction problem and consider the real application to generalize the benefits of N models in designing the networks.

## Introduction

Geodetic networks are indispensable for most engineering projects such as mining and construction, also they are important for studying natural events such as crustal movements. Depending on its stable and identifiable points positioned on the Earth’s surface or close to it and associated with a known coordinate reference system, the geodetic network can be used for monitoring, implementation, establishment, and maintenance purposes. To achieve these purposes, the geodetic network should be designed in such a way that satisfies the requirements of each purpose that contains precision, reliability, and cost.

Four orders were stated by Grafarend^1^ as a solution for the design problem. In zero-order design, the suitable datum is determined for the network; the first-order design calculates the adequate configuration for the network; the second-order design selects the type and weight of the observables; improving the network by densification or expansion is performed in the third-order design. Because each order is performed independently, incompatibility between the results of these orders can arise. To solve this problem, the optimization technique should be used in the network design^2^. After solving the optimization model, the optimal weights presenting the optimal observables besides the optimal shifts of the initial NPs presenting the optimal configuration of the network are obtained. The mathematical meaning of optimizing the geodetic networks is maximizing or minimizing an objective function that represents the network quality comprising precision, reliability, and economy^3^, which may be subjected to some constraints. Moreover, the optimization model may be single or multi based on the number of objective functions. Norm selection plays an important role in formulating both objective function and constraints and consequently the optimized parameters^3^. The popular norms used in the optimization are L1, L2, and L∞. These norms can be defined as follow: L1 norm—known as the Mean Absolute Error (MAE)—is the sum of the absolute values of a vector; L2 norm—called as Mean Squared Error (MSE)—gives the square root of the sum of the squared values of a vector; L∞—known as the uniform norm—provides the largest absolute value of a vector.

Since the pioneering works of Baarda^4^ and Garafarend^1^, a series of investigations and developments in this seminal field were released as published papers in scientific journals. In this context, Kuang^3^ has presented Single and Multi-Objective Optimization Models, (SOOM) and (MOOM), to optimize the control and deformation monitoring networks. SOOMs concern optimizing networks based on either maximum precision, maximum reliability, or low cost. While MOOMs are used for optimizing more than one criterion. Most studies followed this study used and modified these models to design the geodetic networks based on different targets. For instance, Dare and Saleh^5^ used optimization to reduce the cost of performing an epoch survey, which is required to monitor network observed using GPS, consisting of several observing sessions by calculating the cheapest session schedules. They found that their optimization technique is suitable only for small networks. Seemkooei^6^ designed a geodetic network based on maximum reliability and introduced the relationship between the reliability and robustness of the network. It is found that the observations having minimum redundancy numbers lead to the greatest robustness parameters. He concluded that the network can be designed in sense of maximum reliability and checked by robustness to produce a network with high strength. The same author^7^ suggested a new method to design the geodetic networks in SOD considering maximum reliability. His method depends on improving the weight of the observations in such a way that all observations get the same redundancy numbers. To find the most suitable SOOM for designing the geodetic network, Eshagh, and Kiamehr^2^; Bagherbandi et al.^8^; and Alizadeh-Khameneh et al.^9^ made a comparison between the results obtained by these models in different studies. The results of all studies showed the superiority of SOOM of reliability in designing the geodetic networks optimally. The variances criterion that related the sum of total station coordinates and orientations was used by Alizadeh-Khameneh et al.^10^ to determine the optimal position of the total station. The results reported that the height component has an insignificant effect on determining the vertical position of the total station. Most of the aforementioned studies using SOOMs reported that there exist some cases facing inconsistency between constraints such as inconsistencies between cost and precision. Where these inconsistencies can lead to unacceptable results^2^. This problem motivated researchers to use MOOMs instead SOOMs. In this regard, Mehrabi^11^ used a Bi-Objective Optimization Model (BOOM) in optimizing the geodetic network considering maximum precision and reliability. Also, Bagherbandi et al.^8^ (in the same aforementioned study) performed a comparison between SOOMs and MOOMs and found that MOOMs provide the most suitable solution to overcome contradictions between constraints. The capability of the BOOMs versus SOOMs was introduced in Eshagh and Alizadeh-Khameneh^12^. The study showed that the unconstrained BOOM of reliability and precision is the best model, by which the precision and reliability demand can be fulfilled. Moreover, this model is also economical where more observables are eliminated from the observation plan whilst adding the constraints leads to saving more insignificant observables. MOOMs were applied to the same example studied by Alizadeh-Khameneh et al.^9^ to investigate their ability in designing monitoring networks. They found that all quality requirements of the network were met by about 17% better than the initial observation plane, which means that time, cost, and efforts were saved by about 17%. As a summary for this part, these studies demonstrate the capability of MOOMs to solve the problem of contradiction between constraints that existed in SOOMs. This means that MOOMs can design precise, reliable, and low-cost networks at the same time. Some recent studies used diverse methods and procedures to modify the existing optimization technique. For example, Kobry^13^ presented a new approach to design geodetic networks accounting for different quality criteria. The new approach depends on designing the geodetic network using the Multi-Criteria Decision Making (MCDM) methods. Alizadeh-Khameneh et al.^14^ used different observation methodologies to study the uncertainty of tunnel surveying networks. They found that the uncertainty and reliability of the network can be improved by inserting more free station setups and including observations from the points of the tunnel wall bracket. Moreover, the network precision can be improved significantly when gyro data is used. A new computer simulation method aiming for low observation cost and maintaining reliability and accuracy was introduced by Postek^15^ (see also Rofatto et al.^16^; Klein et al.^17^; Matsuoka et al.^18^; Pertusini et al.^19^; and Kobry^13^. In recent years, some modern heuristic techniques were used to solve complex problems that are hard to solve by the traditional techniques. Heuristic techniques depend on gradual improvement in quality criteria until the desired design requirement is fulfilled^20^. However, such techniques do not constantly assure the convergence to a global optimum moreover they are usually stuck in the local minima or maxima, based on the conventions followed^21^. Global optimization systems, such as simulated annealing (SA), genetic algorithm (GA), and particle swarm optimization (PSO), are desired to avoid local minima^22^. In the last three decades, numerous evolutionary optimization algorithms have been helped in solving complex optimization problems, which imitate the process of natural progression in the direction of the solution of the optimization problem. Here some studies used these techniques: GA (Haupt and Haupt^23^; and Doma and Sedeek^24^), PSO (Eberhart and Kennedy^25^; Engelbrecht^26^; Banks et al.^27^), and SA (Azencott^28^; Berné and Baselga^29^; and Odziemczyk^30^).

As far as known, no study has investigated the feasibility of designing the geodetic network in such a way be close to the Project Border (PB). PB term represents the outer boundaries of the work area of the engineering project. For example, in mining and quarrying projects, the work area contains the locations of prospecting; drilling; blasting; exploiting; loading; stocking; explosives warehouse; etc. As logically known, locating Net Points (NPs) near the PB leads to reducing the length of the distances connecting either NPs or those distances connecting the NPs with the Target Points (TPs) located inside the PB. These short distances affect positively the precision of the NPs^31^. Moreover, short sight is less susceptible to the dramatic changes of natural errors, which change from point to point along the line of sight. Wind speed, air temperature, atmospheric pressure, humidity, gravity, and atmospheric refraction are examples of such natural error sources^3^. Besides, the earth curvature error proportion directly with the sight distance. Human errors appearing due to the lack of hearing and vision abilities during the measuring process become minimum in case of short sight. All these errors can degrade the accuracy of the observations and decrease communication between workers when the sight distance is long. Also, short distances reduce transportation costs along these distances. To determine the ideal locations for the NPs near PB, the distances between NPs and PB should be minimized based on optimization technique. This is because the optimization technique not only locates the NPs beside the PB but also provides the optimal configuration, by which the precision of the network can be improved. Moreover, optimization avoids locating the NPs in dangerous areas, the work area. In contrast, simply fixing of NPs near PB without optimization requires to try and error technique to attain the suitable configuration, which leads to a suboptimal solution, consuming time and effort, and increasing the design cost.

This paper provides a novel quality criterion to design the horizontal network in terms of Neighboring (N). The proposed criterion measures how the network is close to PB. The optimization of the geodetic network based on the N criterion seeks to minimize the distances between NPs and the PB. Consequently, all distances will be shorter, then the quality of the network and accuracy of the observations, and cost of the transportation can be improved. The article is prepared into sections devoted to a completed discussion for optimization of the geodetic network based on the suggested criterion. The next section describes the optimization solution based on N criteria. The results and discussions illustrated by a numerical example are introduced in the third section. The fourth section contains the important findings and conclusions.

## Research method

### Contribution and objectives

This study suggests a new criterion in terms of N to optimize the geodetic networks, by which both of quality of the network and accuracy of observations can be improved besides the transportation cost between points can be decreased. The main objectives of this study are listed below:

1. To derive the N criteria to be used as an objective function
2. To formulate N optimization models based on different approximation norms
3. To demonstrate the feasibility of N optimization models based on a numerical example
4. To validate the efficiency of the N criterion

**Figure 1:**
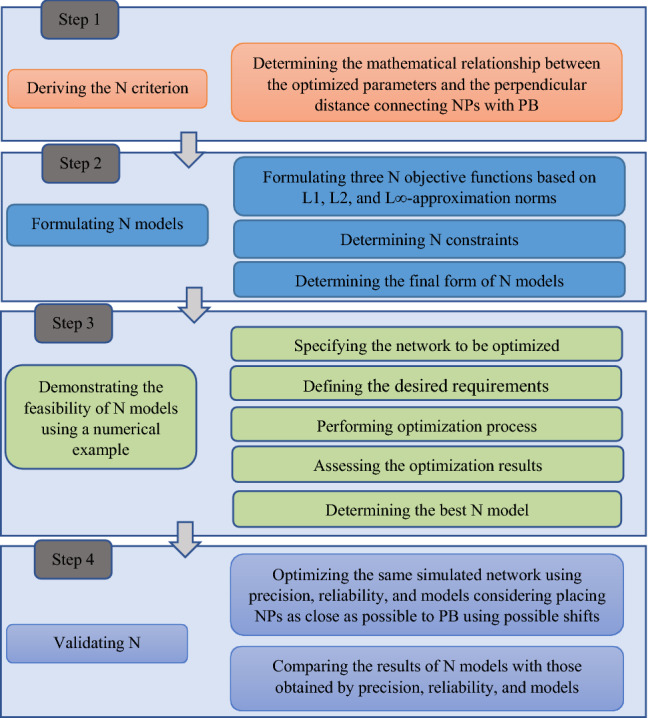
Study scheme.

The study was conducted by the scheme shown in Fig. 1. It presents four steps to perform the research target. Step 1 derives the N criterion by writing the distances linking the NPs with PB in a mathematical form comprising parameters to be optimized. Step 2 is to formulate N optimization models based on three objective functions normalized by L1, L2, and L∞-approximation norms then subject each objective function to safety, precision, reliability, and cost constraints. Step 3 uses N models to optimize a simulated horizontal network to ensure the feasibility of the proposed criteria and determine which norm should be used to formulate the N objective function leading to the best design. Step 4 concerns the validation of the N models by comparing the results released by the N model with those derived by basic models, precision; reliability; and cost models.

### Derivation of N criterion

To find the optimal design of a geodetic network, certain changes must be added to the initial positions and observation weights. To attain an analytical technique to perform the network optimization, the changes of the initial NPs and the observation weights must be introduced by mathematical terms. Let us assume a network in two‒dimensional space with *m* NPs having approximate coordinates of $(x_{i}^{0} ,y_{i}^{0} )$, *i* = 1…*m*, and *n* observations having approximate weights of $p_{l}^{0}$, *l* = 1…n. Assuming the changes in the approximate coordinates and initial weights are $(\Delta x_{i} { },\Delta y_{i} ,\Delta p_{l} ).{ }^{ }$ Thus, the optimized positions and weights can be calculated according to the following formulas^3^

1 $$\begin{aligned} & x_{i} = x_{i}^{0} + \Delta x_{i} \\ & y_{i} = y_{i}^{0} + \Delta y_{i} \\ \end{aligned}$$

and

2 $$p_{l} = p_{l}^{0} + \Delta p_{l}$$

This solution for position changes and observation weights becomes optimal if it fulfills the optimality criterion defining the quality of the network. At this point, the main problem is bringing the quality criterion into a strong mathematical form establishing the relationship that connects the quality criterion with the unknown parameters to be optimized, $(\Delta x_{i} ,\Delta yi,\Delta p_{l} ).$ This step can be done using linearization of the nonlinear matrix equations correlated to network design, which alters the network quality requirement into restrictions on the unknown parameters.

**Figure 2:**
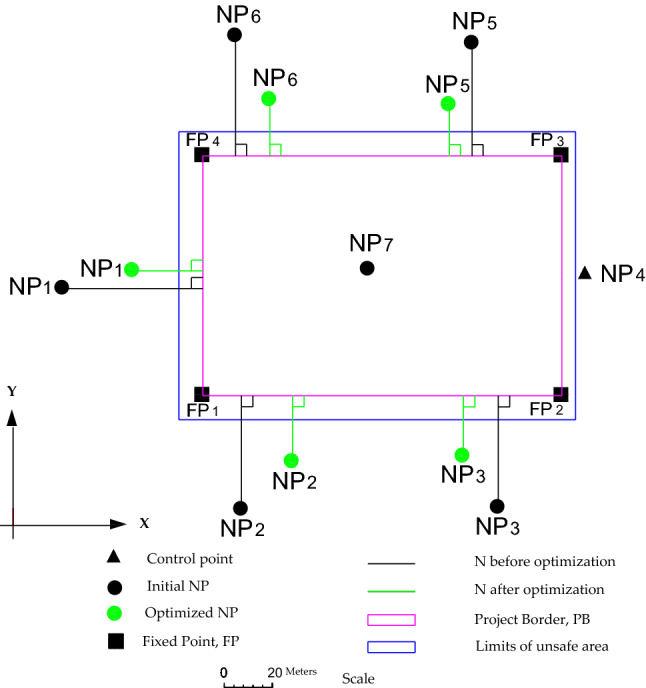
N of NPs before and after optimization. The lines connecting the NPs were canceled for simplification.

In this paper, the N quality criterion is considered to optimize the geodetic network in two‒dimensional space. As mentioned in the introduction section, the N criterion measures how NP is close to the PB. So, it represents the distance between NPs and PB, as shown in Fig. 2. Here, the perpendicular distance should be considered because it represents the shortest distance. Assuming that PB is defined by k Fixed Points (FPs) having coordinates $({\mathbf{x}}_{j} , {\mathbf{y}}_{j} )$, *j* = 1…k. For example, rectangular PB is defined by four FPs (k = 4). To calculate the perpendicular distance from any NP to the facing border side, the linear equation for this border side should be formulated first. This linear equation can be formulated by any two FPs located on this border side as

3 $$a{\mathbf{x}} + b{\mathbf{y}} + c = 0$$

where a, b, and c are the coefficients of the linear equation of a specific border side. Accordingly, the N value for each NP can be calculated by the following common formula that calculates the distance from a point to a line

4 $${\text{N}}_{i} = \frac{{\left| {ax_{i} + by_{i} + c} \right|}}{{\sqrt {a^{2} + b^{2} } }}$$

${\text{N}}_{i}$ stands for the N value of an initial NP $(x_{i}^{0} ,y_{i}^{0} )$; a, b, and c are defined as before. It should be noted that the number of N in the network, either before or after optimization, equals the number of NPs even control points like NP4 or those NPs already located inside the PB like NP7, respectively. The possible shifts can be used to exclude any NP.

The next step is formulating the distance N in such a way comprising $\Delta x_{i} ,\Delta y_{i} ,\;{\text{and}}\;\Delta p_{l} .$ As mentioned before this step can be done using the Taylor linearization series as follows

5 $${\text{N}} = {\text{N}}^{0} + \mathop \sum \limits_{1}^{m} \left( {\frac{{\partial {\text{N}}}}{{\partial x_{i} }}} \right)\Delta x_{i} + \mathop \sum \limits_{1}^{m} \left( {\frac{{\partial {\text{N}}}}{{\partial y_{i} }}} \right)\Delta y_{i}$$

with ${\text{N}}^{0}$ is the vector of all Ns calculated at the approximate values of the network coordinates;${ }\frac{{\partial {\text{N}}}}{{\partial x_{i} }}{\text{ and}}\frac{{\partial {\text{N}}}}{{\partial y_{i} }}$ are the differentiation of the N concerning the coordinates of the NPs; $\Delta x_{i} ,{\text{ and }}\Delta y_{i}$ are defined as before. Now, the N criterion can be written as follows

6 $${\text{N}} = {\text{N}}^{0} + {\text{N}}_{1} {*}w$$

where

7 $${\text{N}}_{1} = \left[ {vec\left( {\frac{\partial N}{{\partial x_{1} }}} \right)vec\left( {\frac{\partial N}{{\partial y_{1} }}} \right) \ldots vec\left( {\frac{\partial N}{{\partial x_{m} }}} \right)vec\left( {\frac{\partial N}{{\partial y_{m} }}} \right)\;{\mathbf{0}}} \right]$$

8 $$w = (\Delta x_{i} \;\Delta y_{i} \ldots \Delta x_{m} \;\Delta y_{m} \;\Delta p_{1} \ldots \Delta p_{n} )^{T}$$

$w$ is (2* m* + *n*) by one vector; $\Delta p_{1} \ldots \Delta p_{n}$, $m,\;{\text{and}}\;n$ are introduced before; ${\text{N}}_{1}$ is $m\;by\;\left( {2m + n} \right)$ matrix; $v{\text{e}}c$ is an operator that produces a vector by stacking the columns of a quadratic matrix one under another in a single column. 0 is $m\;by\;n$ submatrix of zeros corresponding to the weight changes.

### Formulating the N models-SOOMs of N

#### Objective functions

After deriving the N criterion and introducing it as a function in the parameters to be optimized (Eq. 6), the N objective function should be normalized by L2, L1, and L∞-norms, ${\text{N}}_{{{\text{L}}1,{\text{L}}2,{\text{L}}\infty }}$. This is needed to find the best norm that can fit the N criterion and then provide the optimal solution. Where the norm choice controls the final solution of the optimization model^3^. This means three objective functions can be obtained. Accordingly, the simplified form for minimizing the N objective functions can be written as

9 $$\left\| {\text{N}} \right\|_{{{\text{L}}1,{\text{L}}2,{\text{L}}\infty }} = {\text{min}}\;\left( {{\text{optimal}}\;{\text{N}}} \right)$$

The minimized values of N should be controlled by preset values to protect the NPs from destruction (safety constraint) due to falling in the danger area (blue line in Fig. 2) as follows

10 $$\left\| {\text{N}} \right\|_{{{\text{L}}1,{\text{L}}2,{\text{L}}\infty }} \ge {\text{n}}_{m} \;\left( {{\text{N}}\;{\text{control-safety}}\;{\text{condition}}} \right)$$

where N is the vector consisting of all initial N values; ${\text{n}}_{m}$ is the vector comprising the preset values of safety requirements; *m* is the number of Ns that equals the total number of NPs;$\left\| . \right\|$ is the vector norm. For more details about norms, the reader can see Kuang^3^.

#### Constraints

To ensure that the designed network satisfies the desired requirements, N objective functions should be subjected to the desired precision, reliability, and cost besides physical constraints. The following mentions these constraints in a brief, where the details can be found in Kuang^3^.

##### Precision constraint

The precision of the NPs is the most important demand in the designed network. Where the geodetic network should deliver precision not less than the client requirements. Accordingly, this constraint must be considered during the design process. The precision constraint intends to make the solution of the configuration matrix $A$ and the weight matrix $P$ yield a variance–covariance matrix $C_{x}$ of the coordinates in some sense at least equal to or better than the proposed criterion matrix $C_{s}$. The precision constraint can be expressed as

11 $$H_{1} w - u_{1} \le 0$$

where

12 $$H_{1} = \left( {I_{u} \Theta I_{u} } \right)^{T} \;H;\; u_{1} = \left( {I_{u} \Theta I_{u} } \right)^{T} \;u;\;{\text{and}}\;u = vec\left( {C_{s}^{0} } \right) - vec\left( {C_{x}^{0} } \right)$$

13 $$H = \left[ {v{\text{e}}c\left( {\frac{{\partial C_{x} }}{{\partial x_{1} }} - \frac{{\partial C_{s} }}{{\partial x_{1} }}} \right)v{\text{e}}c\left( {\frac{{\partial C_{x} }}{{\partial y_{1} }} - \frac{{\partial C_{s} }}{{\partial y_{1} }}} \right) \ldots v{\text{e}}c\left( {\frac{{\partial C_{x} }}{{\partial x_{m} }} - \frac{{\partial C_{s} }}{{\partial x_{m} }}} \right)v{\text{e}}c\left( {\frac{{\partial C_{x} }}{{\partial y_{m} }} - \frac{{\partial C_{s} }}{{\partial y_{m} }}} \right)v{\text{e}}c\left( {\frac{{\partial C_{x} }}{{\partial p_{1} }}} \right) \ldots { }v{\text{e}}c\left( {\frac{{\partial C_{x} }}{{\partial p_{n} }}} \right)} \right]$$

14 $$C_{x} = \sigma_{o}^{2} Q_{x} = \sigma_{0}^{2} \left[ {\left( {A^{T} PA + DD^{T} } \right)^{ - 1} - H \left( {H^{T} DD^{T} H} \right)^{ - 1} H^{T} } \right]$$

where

15 $$A = \frac{\partial f}{{\partial X}};\;P = \sigma_{0}^{2} C_{l}^{ - 1} ;\;C_{s} = SC_{TK} S^{T} ;\;{\text{and}}\;S = \left[ {I - H\left( {D^{T} H} \right)^{ - 1} D^{T} } \right]$$

the configuration matrix A is *n* by 2* m* and consists of the partial derivatives of the parametric equations of the observations (*f*) concerning unknown coordinates (*X*). The weight matrix *P* is *n* diagonal matrix, $\sigma_{0}^{2}$ (mm) is the prior variance factor taken equals 1 in the design stage and $C_{l}^{ }$ stands for the variance–covariance matrix of the observation. The criterion matrix $C_{s} { }$ is a 2* m* covariance matrix representing the desired precision of the optimized coordinates. ${ }C_{TK}^{ }$ is Taylor‒Karman criterion matrix. $D^{ }$ and $H$ are the minimum constrained and its agreeing inner constrained datum matrices. $I_{u}$ is *u* unit matrix; $\Theta$ introduces the Khatri‒Rao product; $C_{x}^{0}$ and $C_{s}^{0}$ are $C_{x}$ and $C_{s}$, respectively, which are calculated at the approximated values of the coordinates and weights.

##### Reliability constraint

Similar to precision, enough reliability for the geodetic network is essential to detect gross errors and diminish the effects of those hidden gross errors on the coordinates of the network. To do so, the designer should consider the required reliability. The reliability constraint tries to make the solution of the configuration matrix $A$ and the observation weight matrix $P$ produce a network with redundancy numbers $r_{ }$ equal to or greater than the required one $r_{m}$. The reliability constraint can be introduced as

16 $$\left\| {r_{00} - R_{11} \;w} \right\| \ge r_{m}$$

where

17 $$r_{00} = \left( {I_{n} \Theta I_{n} } \right)^{T} \;r_{0} ;\;r_{0} = vec\left( {R^{0} } \right);\;{\text{and}}\;R_{11} = \left( {I_{n} \Theta I_{n} } \right)^{T} R_{1}$$

18 $$R_{1} = \left[ {v{\text{e}}c\left( {\frac{{\partial {\text{R}}}}{{\partial x_{1} }}} \right)v{\text{e}}c\left( {\frac{\partial R}{{\partial y_{1} }}} \right) \ldots v{\text{e}}c\left( {\frac{{\partial {\text{R}}}}{{\partial x_{m} }}} \right)v{\text{e}}c\left( {\frac{{\partial {\text{R}}}}{{\partial y_{m} }}} \right)v{\text{e}}c\left( {\frac{{\partial {\text{R}}}}{{\partial p_{1} }}} \right)v{\text{e}}c\left( {\frac{{\partial {\text{R}}}}{{\partial p_{n} }}} \right)} \right]$$

19 $$R = I - A\left( {A^{T} PA + DD^{T} } \right)^{ - 1} A^{T} P$$

$I_{n}$ is *n* unit matrix; and $R_{ }^{0}$ is the matrix of redundancy numbers $R_{{}}^{{}}$, which are calculated at the approximated values of the coordinates and weights.

##### Cost constraint

The cost of the geodetic network is the third most important criterion affecting the network design and the most difficult criterion to formulate mathematically. The most approximate approach to formulate the cost criterion mathematically is dividing the costs for measurements as constant terms. For example, the cost of transportation between the stations, setting up the instruments and signaling the points. Considering that the small weight of an observation marks it as less expensive, the approximated mathematical formula of the cost criterion can be introduced as

20 $$\left\| {\varvec{P}} \right\| = {\text{min}}{.}$$

The cost constraint intends to make the solution of the weight matrix $P$ satisfy or less than the preset cost $c_{m}$ for the network. The cost constraint can be formulated as

21 $$\left\| {c_{00} + C_{11} w} \right\| \le c_{m}$$

where

22 $$C_{11} = \left( {I_{n} \Theta I_{n} } \right)^{T} \;C_{1} ;\; c_{00} = \left( {I_{n} \Theta I_{n} } \right)^{T} \;c_{0} ;\;{\text{and}}\;c_{0} = vec\left( {P^{0} } \right)$$

23 $$C_{1} = \left[ {{\mathbf{0}}\;v{\text{e}}c\left( {\frac{\partial P}{{\partial p_{1} }}} \right)v{\text{e}}c\left( {\frac{\partial P}{{\partial p_{n} }}} \right)} \right]$$

**0** is a submatrix of zeros that belongs to the position shifts, the other terms are defined as before.

##### Physical constraints

- Datum consideration

To prevent the network shape from changing due to rotation, scaling, and differential rotation, NPs shifts should be controlled. In the case of horizontal networks, fixing the coordinates of a specific NP and fixing the azimuth and distance from this NP to another one provides the following constraint equation

24 $$\left( {D^{T} \;{\mathbf{0}}} \right)w = {\mathbf{0}}$$

**0** is zero submatrices related to the weight improvements. $D^{T}$ is the datum matrix (4 × 2* m*), based on the measured observations, the related rows may be deleted. For example, the first two rows are deleted if the coordinates of an NP are measured. The third row is deleted if an azimuth is measured. While the fourth row is deleted if distances are measured.

- Realizability

Field obstacles such as topography should be considered to introduce the possible shifts for the NPs. Also, the weights of observations are required to be positive and limited by the highest achievable accuracy of the existing instruments. The following equations show the shift and weight constraints.

25 $$a_{1i} \le \Delta x_{i} \le a_{2i} ;\;{\text{and}}\;b_{1i} \le \Delta y_{i} \le b_{2i} \;\left( {i = 1, \ldots m} \right)$$

26 $$0 \le p_{l} \le \frac{{\sigma_{o}^{2} }}{{\left( {\sigma_{l} } \right)_{min}^{2} }}\;{\text{or}}\; - p_{l}^{0} \le \Delta p_{l} \le \frac{{\sigma_{o}^{2} }}{{\left( {\sigma_{l} } \right)_{min}^{2} }} - p_{l}^{0} = \left( {\Delta p_{l} } \right)_{max} \;\left( {l = {1}, \ldots n} \right)$$

$[a_{1i} ,a_{2i} ]$ and $[b_{1i} ,b_{2i} ]$ are the shift limits for the NPs; and $\left( {\sigma_{l} } \right)_{min}^{2}$ is the least variance that can be performed for each observable^3^. Combining Eqs. (25) and (26), the following new equation can be obtained

27 $$A_{00} { }w \le b_{00}$$

where

28 $$A_{00} = \left[ {\begin{array}{*{20}c} I \\ { - I} \\ \end{array} } \right];\;b_{00} = \left[ {a_{21} b_{21} \cdots a_{{2{\text{m}}}} b_{{2{\text{m }}}} \left( {\Delta p_{1} } \right)_{max} \cdots \left( {\Delta p_{n} } \right)_{max} - a_{{11{ }}} - b_{{11{ }}} \cdots - a_{{1{\text{m}}}} - b_{{1{\text{m}}}} p_{1}^{0} \cdots p_{n}^{0} } \right]{ }^{T}$$

$I$ is a unit matrix with dimensions of (2* m* + *n*) by (2* m* + *n*).

#### SOOMs of N

After formulating three N objective functions based on different norms and determining the constraints controlling the design results, three SOOMs for N were formulated namely, NL1, NL2, and NL∞ to determine which model can provide the best solution. The general mathematical form for the N models is presented by the following equations. Equation (29) shows three objective functions based on L2, L1, and L∞ norms. The safety constraint presented in equation (30) is normalized by the same norm used in the objective function in the same model. The precision constraint is shown in equation (31). Regardless of the model used, L∞ and L1 norms were used as the best fitting norms for the reliability, and cost constraints introduced in Eqs. (32) and (33), respectively, as reported by Kuang^3^. Datum constraints and realizability presented in Eqs. (34) and (35) are the same for all models. It should be noted here that the N models can be applied to all horizontal networks measured by either correlated or uncorrelated observations.

29 $$\left\| {{\text{N}}_{1} {*}w + {\text{N}}^{0} } \right\|_{{L1,L2,L\infty { }}} = {\text{min}}.$$

Subject to:

30 $$\left\| {{\text{N}}_{1} {*}w + {\text{N}}^{0} } \right\|_{L1,L2,L\infty } \ge {\text{n}}_{{\text{m}}}$$

31 $${\text{H}}_{1} {*}w - {\text{u}}_{1} \le 0$$

32 $$\left\| {{\text{R}}_{11} {*}w + {\text{ r}}_{00} } \right\|_{L\infty } \ge {\text{r}}_{{\text{m}}}$$

33 $$\left\| {{\text{C}}_{11} {*}w + {\text{c}}_{00} } \right\|_{L1} \le {\text{c}}_{{\text{m}}}$$

34 $$\left( {D^{T} \;{\mathbf{0}}} \right)w = {\mathbf{0}}$$

35 $$A_{00} \;w \le b_{00} .$$

## Results and discussion

### Numerical example

**Figure 3:**
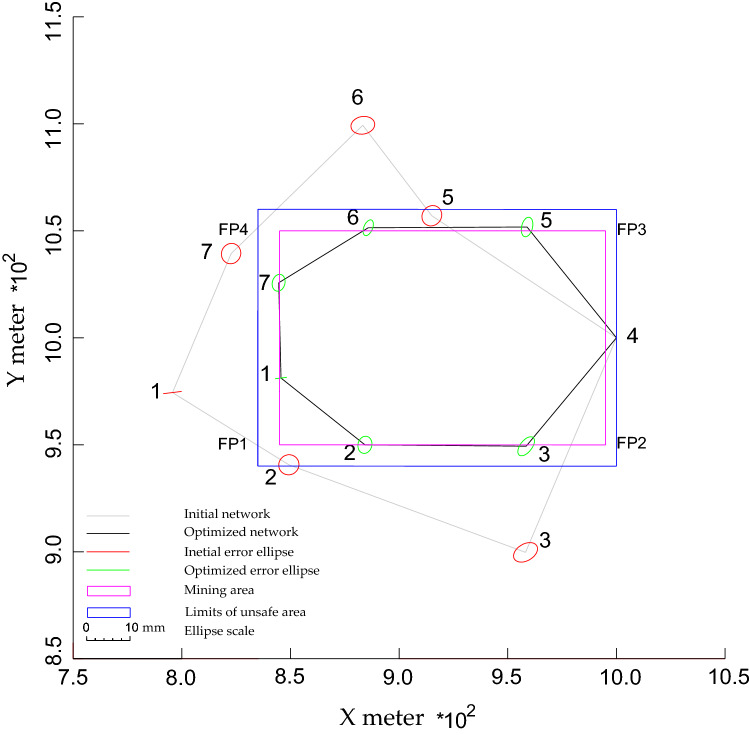
Optimized configuration of the geodetic network based on NL1.

To study the capability of N models in designing the horizontal geodetic networks and its feasibility in improving the quality of the network and accuracy of the observations and decreasing the transportation cost, a simulated horizontal trilateration geodetic network was considered (see Fig. 3). It is assumed that this network serves a mining area surrounded by a rectangle border (purple border). This border is defined by four FPs, which are located at the corners. The network consists of 7 NPs connected by 21 distances having equal initial weights of 0.1. The approximated positions of NPs 2 and 5 are inside the unsafe area, which is marked as a blue rectangle and separated by 10 meters from the mining area (purple border). The reason here is to determine the ability of the N models to arrange these two NPs outside the unsafe area. While the remaining NPs were positioned outside the unsafe limits. NP 4 and the direction from NP 4 to NP1were kept fixed as the optimal datum of the network. It is supposed that the optimization process should be done in such a way that the precision of NPs ≤ 2mm and the redundancy number for each distance ≥ 0. 4. The optimized weights are supposed to be positive and ≤ 1/(2 mm)^2^. The N value for each NPs should be ≥ 10 m to be safe from destruction (outside the blue border). In this example, realizability (Eq. 35) was not applied. This is to determine the capability of the N model to find the best configuration.

Two targets should be realized to demonstrate the efficiency of the N models. The first target is the ability of N models to improve the quality of the observations and decrease the transportation cost between points. This target can be achieved if all NPs optimized very close to the PB outside the unsafe area to produce short distances, which can be concluded from N results. The second target is the ability of N models to improve the quality of the network itself. In other words, to design the geodetic network in such a way satisfying safety, precision, reliability, and cost requirements. Besides these two targets, it is required to determine which N mode can satisfy these two targets.

**Figure 4:**
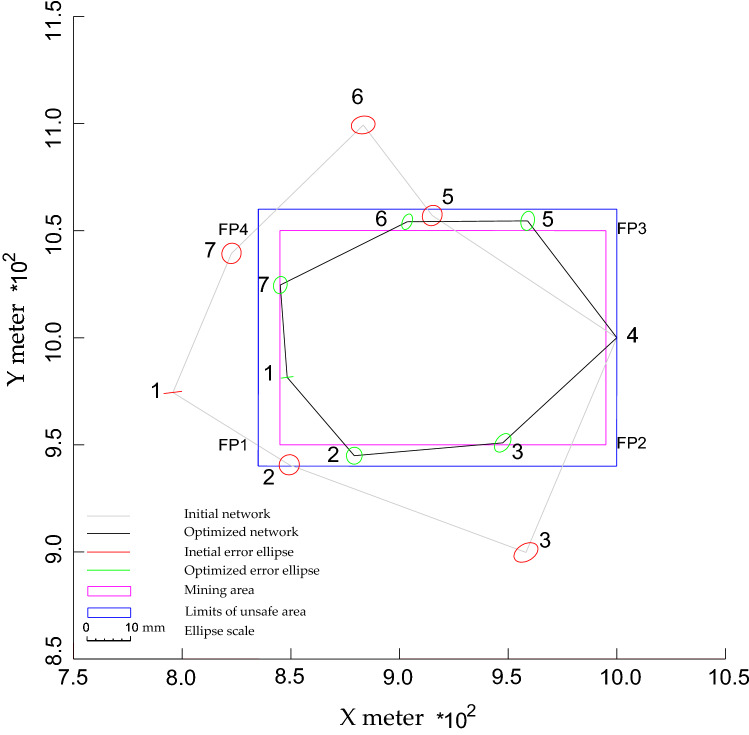
Optimized configuration of the geodetic network based on NL2.

**Figure 5:**
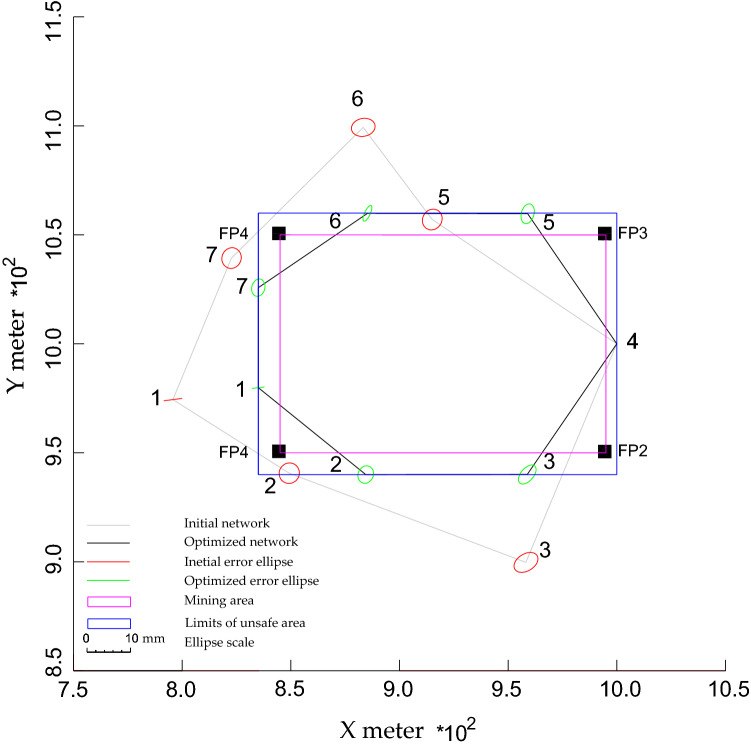
Optimized configuration of the geodetic network based on NL∞.

**Figure 6:**
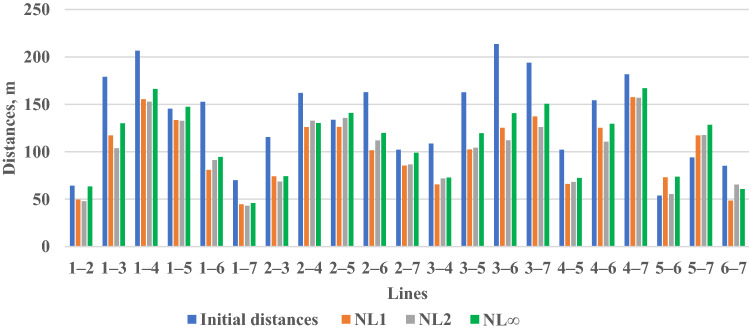
Initial and optimized distances between NPs based on different N models.

For the first target, improving the accuracy of the observations and transportation cost: the results delivered by N models showed that all NPs become closer to the PB after optimization as shown in Figs. 3, 4, and 5 and Table 1. Table 1 shows that the minimum N values were produced by NL1 and NL2, while NL∞ provided the greatest values. These values led to creating short distances between points as shown in Fig. 6. It shows that the initial distances decreased by ~ ¼ % (from 135 to 104 m, on average). These results demonstrate the ability of all N models in improving the observations accuracy and transportation cost with superiority for NL∞ because it relocated NPs at safe positions.

**Table 1:** Results of N before and after optimization depending on different N models: Unit: m.

Pt	Before optimization	After optimization		
		NL1	NL2	NL∞
1	50	− 1	− 3	10
2	9	0	5	10
3	50	1	− 1	10
4	5	5	5	5
5	7	2	5	10
6	50	1	4	10
7	22	0	0	10
Mean	28	1	2	10

The means and the standard deviations do not comprise NP 4.

For the second target, satisfying the quality of the network: due to minimization of the initial N values, all models minimized initial N significantly as shown before in Table 1. However, only NL∞ can provide the ideal results satisfying the safety requirements. Where all NPs produced by this model were replaced at safe positions with similar N values of 10 m, which are equal to the preset safety values. In contrast, the other models failed to relocate all NPs outside the dangerous area. All models provided almost similar improved precision and most of them met the precision demands (see Table 2 and Figs. 3, 4, and 5). This refers to the short distances created between NPs and the control points, 4. This explanation is demonstrated by Alizadeh-Khameneh et al.^9^ and Ghilani.^31^. They stated that the precision of NPs is proportional inversely with the length of distances connecting NPs. This result demonstrates the ability of the N models to satisfy the precision requirements.

**Table 2:** The precision $(C_{x} )$ of NPs before and after optimization based on different N models. Unit: mm.

Precision components	$C_{s}$	$C_{x}$ Before optimization	$C_{x}$ After optimization		
			NL1	NL2	NL∞
$\sigma_{{x_{1} }}$	2	2.1	1.3	1.4	1.4
$\sigma_{{y_{1} }}$	2	0.3	0.2	0.2	0.2
$\sigma_{{x_{2} }}$	2	2.3	1.6	1.8	1.8
$\sigma_{{y_{2} }}$	2	2.3	2.0	2.0	2.0
$\sigma_{{x_{3} }}$	2	2.8	1.9	1.9	2.0
$\sigma_{{y_{3} }}$	2	2.3	2.2	2.2	2.2
$\sigma_{{x_{5} }}$	2	2.3	1.3	1.6	1.5
$\sigma_{{y_{5} }}$	2	2.4	2.3	2.2	2.3
$\sigma_{{x_{6} }}$	2	2.7	0.7	1.2	1.0
$\sigma_{{y_{6} }}$	2	2.1	1.8	1.8	1.8
$\sigma_{{x_{7} }}$	2	2.2	1.5	1.6	1.5
$\sigma_{{y_{7} }}$	2	2.4	2.0	2.0	2.0

Regarding observations cost requirements, the results of optimization showed that all N models failed to eliminate any observable from the design plan as presented in Table 3. This is because that all observations are necessary to satisfy the desired precision. Where it is shown from Table 2 that there are some NPs such as NP 3 and 5 are barely meet the requested precision. Thus, it can be concluded that there is an inconsistency between cost and precision constraints, which is common in SOOMs^2^. Thus, to remove some observables, the desired precision should decrease^12^. Accordingly, it appears that N models have no significant influence on the observation plane, in this example, and consequently on the cost of the observation. Table 3 also shows the reliability results after optimization. It demonstrates that no model succeeded to improve the initial reliability of the network (with an average redundancy number of 0.48). However, the desired reliability (0.4) was satisfied by all models. The reason here is that all observations still working after optimization due that all observations are needed to attain the desired precision. This result points to an inconsistency between reliability and precision constraints.

**Table 3:** The optimized weight (P), accuracy (*σ*), and reliability ( redundancy number, r) that produced by the different N models. Accuracy unit: mm.

Line	P			*σ*			Initial r	Optimized r		
	NL1	NL2	NL∞	NL1	NL2	NL∞		NL1	NL2	NL∞
1‒2	0.21	0.20	0.21	2.2	2.3	2.2	0.46	0.48	0.47	0.47
1‒3	0.22	0.21	0.21	2.2	2.2	2.2	0.47	0.49	0.49	0.49
1‒4	0.23	0.22	0.23	2.1	2.1	2.1	0.55	0.48	0.48	0.47
1‒5	0.21	0.20	0.20	2.2	2.2	2.2	0.48	0.46	0.47	0.47
1‒6	0.20	0.20	0.19	2.3	2.3	2.3	0.47	0.47	0.47	0.46
1‒7	0.18	0.18	0.18	2.3	2.3	2.3	0.47	0.46	0.46	0.46
2‒3	0.21	0.19	0.20	2.2	2.3	2.2	0.47	0.48	0.48	0.48
2‒4	0.21	0.20	0.20	2.2	2.2	2.2	0.45	0.43	0.44	0.44
2‒5	0.17	0.17	0.17	2.4	2.4	2.4	0.44	0.45	0.46	0.45
2‒6	0.18	0.18	0.18	2.4	2.4	2.4	0.51	0.47	0.48	0.47
2‒7	0.19	0.18	0.18	2.3	2.4	2.3	0.47	0.49	0.49	0.49
3‒4	0.16	0.16	0.16	2.5	2.5	2.5	0.28	0.41	0.42	0.42
3‒5	0.18	0.18	0.18	2.3	2.4	2.4	0.50	0.43	0.44	0.44
3‒6	0.23	0.21	0.22	2.1	2.2	2.1	0.56	0.46	0.47	0.47
3‒7	0.20	0.19	0.20	2.2	2.3	2.2	0.51	0.52	0.51	0.52
4‒5	0.23	0.21	0.22	2.1	2.2	2.1	0.52	0.46	0.46	0.46
4‒6	0.23	0.22	0.23	2.1	2.1	2.1	0.45	0.53	0.49	0.53
4‒7	0.21	0.21	0.21	2.2	2.2	2.2	0.52	0.53	0.52	0.52
5‒6	0.20	0.19	0.19	2.3	2.3	2.3	0.51	0.47	0.48	0.48
5‒7	0.20	0.19	0.20	2.2	2.3	2.3	0.48	0.52	0.52	0.52
6‒7	0.19	0.18	0.18	2.3	2.4	2.3	0.45	0.50	0.50	0.49
Mean	0.20	0.19	0.20	2.2	2.3	2.3	0.48	0.48	0.48	0.48

The most important point in this method for practitioners, to get its benefits, is determining the PB. Where the PB should be located at the ends of the work activities. The coordinates of the nodes (FPs) are determined based on any network existing in the field (the precision is not important). Then, the safety distance can be decided to ensure locating NPs outside the dangerous area. Considering NL∞ subjected to the desired safety; precision; reliability; cost, the designer can get a network that satisfies safety and precision requirements, and preserves the initial reliability and observation cost. Besides, the transportation cost between points will discount. To improve the initial reliability and observations cost, the designers are recommended to use MOOMs instead SOOMs as reported by Eshagh and Kiamehr^2^; Bagherbandi et al.^8^; and Alizadeh-Khameneh et al.^9^.

### Validation

There is an objection that may arise against the N models that similar results (minimum network after optimization) can be obtained by maximizing the precision without minimizing the distances between NPs and PB as demonstrated by Berné and Baselga^29^. The response to this is divided into two points based on if the shifts of the NPs are constrained or not.

For the first case, using shift boundaries: it is not inevitable to relocate NPs close to PB and at the same time satisfy the quality of the network at all times. This is because the suitable configuration providing the desired precision may not exist due to shifting limits. Accordingly, the NPs will not obey the shift limits and move to find the configuration providing maximum precision (objective function). In contrast, the N model, which is not subjected to shift limits, seeks to relocate NPs near PB in such a way form a configuration that meets the precision constraint. To demonstrate this, the existing network was redesigned not only in sense of maximum precision but also high reliability and low cost were considered. All three models were subjected to shift boundaries to enforce the NPs to be near the PB. The optimization results listed in Table 4 show that precision, reliability, and cost models positioned NPs far from the PB with average N values of 23, 25, and 19 m, respectively. This result demonstrates the superiority of NL∞ (with N=10 m) over the other models.

**Table 4:** Results of N after optimization depending on precision, reliability, and cost models: Unit: m.

Pt	After optimization With shift limits		
	Precision model	Reliability model	Cost model
1	35	50	10
2	17	19	19
3	27	50	10
4	5	5	5
5	16	17	17
6	50	11	50
7	12	22	22
Mean	23	25	19
Std	15	18	15

The means and the standard deviations do not comprise NP 4.

For the second case, without shift boundaries: due to the absence of shift limits, the NPs may fail inside the danger area because the target of these models is maximizing the precision, or reliability, or reducing the cost. While in the case of NL∞, this cannot be happened due to the safety constraint controlling the position of the NPs.

Although the optimization results demonstrated the feasibility of the N criterion in designing the geodetic networks, there are two major limitations in this study that could be addressed in future research. First, the study focused on SOOMs and did not test the feasibility of MOOMs. Second, a simulated example was considered overlooking the real examples.

As mentioned in the introduction, SOOMs are required to improve only one quality criterion presented as an objective function. This may lead to producing a defective design due to the inconsistency between different criteria as presented in the existing example. Where the reliability and cost constraints were not satisfied due to the contradiction of these constraints with precision constraint/or N criterion. The main motivation to use SOOMs in the present study was to determine the feasibility of the proposed criterion (alone) in designing the geodetic networks. MOOMs can be used as an alternative to overcome this problem as demonstrated by Eshagh and Alizadeh-Khameneh^12^ and Alizadeh-Khameneh^9^. These models use more than one criterion as an objective function and thus all quality requirements can be fulfilled.

The results of the simulated networks can not be generalized because they do not present the field problems, by which the efficiency of the N models can be assessed. For example, the feasibility of N models in improving the accuracy of observations was demonstrated theoretically based on that the short sight is less susceptible to natural errors. This feasibility is required to be demonstrated in common measurements used in real applications such as angles and GPS observations. It should be noted here that this study used a simulated network only to simplify introducing the proposed criterion.

## Conclusions

Optimizing the horizontal geodetic networks considering the distance between Net Points (NPs) and Project Border (PB)‒Neighboring (N) criterion‒was investigated in this paper. N represents the perpendicular distances from NPs to the PB. Minimizing N means relocating NPs near the PB which leads to creating short distances either between NPs or between NPs and Target Points (TPs) to be measured inside PB. As commonly known from the literature, creating short distances after optimization improves the precision of NPs, increases the accuracy of the observations, and decreases the transportation along these distances. So, the presented study aims to improve the quality of the network from one side and improve the quality of observations and reduce the transportation cost between points in real applications from another side based on minimizing distances between NPs and PB.

To be used as an objective function, the N criterion was brought into a solid mathematical form that functions in the parameters to be optimized (NPs shifts and observation weights). Then three N objective functions were formulated based on L1, L2, and L∞ norms to build the corresponding optimization models‒NL1, NL2, and NL∞‒and to determine the best models giving the best design. Each model is subjected to safety, precision, reliability, and cost constraints. Then, a numerical example was considered to demonstrate the efficiency of the proposed criterion. The results are divided into two directions: the first, results demonstrate the feasibility of N models in improving the accuracy of observations and in decreasing the transportation cost between NPs themselves or between NPs and TPs in real applications. The second, results demonstrate the efficiency of N models in improving the quality of the network. For the first direction, the results of N values in all models became minimum. This led to relocating the NPs near the PB and created short distances between all points, due to an average reduction of ¼% in initial value. This means improving the accuracy of observation and transportation cost either in the network or during measuring TPs inside the PB in actual applications. In the case of the second direction, the NL∞ showed superiority in positioning all NPs outside the danger area over the other N models with an ideal N value of 10 m. The precision of the network improved and satisfied the requirements in all models, due to short distances between NPs and the control point. For cost results, no model succeeded to eliminate any observation to reduce the observation cost. Also, all models failed to improve the reliability of the initial network (0.48). The results of cost and reliability point to the inconsistency between these constraints and precision constraint and in turn N criterion. The proposed method was validated by comparing the results of N produced by NL∞ with those produced by precision, reliability, and cost models. The results proved the superiority of NL∞ over the other models in relocating the NPs at safe positions near PB.

In conclusion, the numerical results demonstrated the theoretical feasibility of N models in improving the accuracy of observations and transportation costs between points. Where it is required in future works to prove this feasibility in actual work. Also, the simulated results demonstrated the capability of NL∞ to provide a precise network and preserve the initial reliability and observation cost due to the contradiction of these criteria with the N criterion. To solve this problem, it is recommended to use MOOM instead of SOOMs in future works.
